# The roles of sex and gender in women’s eye health disparities in the United States

**DOI:** 10.1186/s13293-021-00401-3

**Published:** 2021-10-20

**Authors:** Irene O. Aninye, Kathleen Digre, M. Elizabeth Hartnett, Kira Baldonado, Erin M. Shriver, Laura M. Periman, Julie Grutzmacher, Janine A. Clayton, Society for Women’s Health Research Women’s Eye Health Working Group

**Affiliations:** 1 Society for Women’s Health Research, 1025 Connecticut Avenue, NW, Suite 1104, Washington, DC 20036 USA; 2 Ophthalmology and Visual Sciences, University of Utah, Salt Lake City, UT USA; 3 Prevent Blindness, Chicago, IL USA; 4 Ophthalmology and Visual Sciences, University of Iowa, Iowa City, IA USA; 5 Periman Eye Institute, Seattle, WA USA; 6 Office of Research on Women’s Health, National Institute of Health, Bethesda, MD USA

**Keywords:** Dry eye, Retinopathy, Gender inequity, Health disparities, Macular degeneration, Telehealth, Patient and provider education, Population health, Thyroid eye disease, Vision loss

## Abstract

**Background:**

In the United States, women are at a higher risk of developing vision impairment or a serious eye disease (such as age-related macular degeneration, thyroid eye disease, or chronic dry eye disease) than men. Disparities in eye diseases due to biology widen even further when considering factors such as social determinants of health; gaps in research data, literature, and policy; insufficient provider and patient education; and limitations in screening and treatment options. Sex and gender disparities in eye health are clinically under-addressed and burdensome on both patient quality of life and the health care and economic systems, resulting in a pressing population health issue that negatively impacts women.

**Design:**

The Society for Women’s Health Research convened a working group of expert clinicians, researchers, and patient advocates to review the current state of science regarding sex and gender disparities in women’s eye health, identify knowledge gaps and unmet needs, and explore better means to advance research, improve patient care, and raise awareness of key issues.

**Discussion:**

The SWHR Women’s Eye Health Working Group identified priority areas in research, clinical care, and education to reduce disparities and improve patient care in women’s eye health. The working group recommends using a systems approach that incorporates a comprehensive research framework with a sex and gender lens to guide future work and that increases health care provider and public education, as well as engagement by expanding partnerships among ophthalmologic providers, researchers, and non-vision stakeholders.

## Background

According to the National Center for Chronic Disease Prevention and Health Promotion, approximately 12 million Americans ages 40 and older are visually impaired or blind, and more than half of them are women [1]. The most prevalent eye diseases in women are age-related macular degeneration, glaucoma, and cataracts. With increases in life expectancy, diabetes, and other chronic diseases, incidence of eye diseases is expected to double by the year 2050 [2]. Respondents in a nationwide poll (comprising 52% women) about attitudes toward eye health reported vision loss as equal to or worse than losing memory, speech, hearing, or a limb [3]. Investigators found that the highest concerns for respondents were the impacts on quality of life and loss of independence. Although many causes of vision impairment are preventable or treatable, eye health is often not prioritized in research, policy, or public health measures to ensure optimal outcomes.

Research often fails to consider the roles of sex and gender in eye health, leaving gaps in our understanding and ability to address differences in patient populations and outcomes. Sex is an important biological variable that can influence the function of biological processes in males and females, as well as how individuals will respond to medications for disease [4]. Gender—a person’s self-representation in response to social and environmental experiences—can play an equally influential role in health and disease. A 2015 review by Clayton and Davis [5] examined how sex and gender disparities relate to women’s eye health across disease states. The review served as a foundation for a roundtable meeting dedicated to understanding women’s eye health across the lifespan and prioritizing areas to address knowledge gaps and unmet needs in the field. This paper expands upon their original analysis and discusses solutions to reduce the persisting gaps in eye health due to sex- and gender-specific biological factors and social determinants of health.

## Methods

The Society for Women’s Health Research (SWHR) assembled a Women’s Eye Health Working Group of researchers, clinicians, and patient advocates to: (1) assess the state of the science regarding sex and gender differences in women’s eye health across the lifespan; (2) examine a spectrum of vision disorders that disproportionately or exclusively impact women to identify key influences and determinants (e.g., biological, behavioral, and societal) for these disparities; and (3) develop recommendations to advance the research agenda, diagnostic and treatment strategies and to address gaps in awareness, education, and stakeholder engagement. SWHR selected 12 participants with expertise in women’s eye health and research interests in sex differences in disease manifestation and symptoms to participate in a 2-day roundtable meeting (Table 1). The experts also represented diversity in training, background, gender, and geographic location.

**Table 1:** Society for Women’s Health Research Women’s Eye Health Roundtable Participants

Kira Baldonado, MPH, Vice President of Public Health and Policy, Prevent Blindness	
Emily Y. Chew, MD, Director of the Division of Epidemiology and Clinical Applications, National Eye Institute	
Janine A. Clayton, MD, Director of the Office of Research on Women’s Health, National Institute of Health	
Kathleen Digre, MD, Distinguished Professor of Neurology, Professor of Ophthalmology and Visual Sciences, Moran Eye Center, University of Utah	
Tamara R. Fountain, MD, Professor of Ophthalmology, Rush University Medical Center	
Lynn K. Gordon, MD, PhD, Professor Emeritus of Ophthalmology, Stein Eye Institute, David Geffen School of Medicine at University of California, Los Angeles	
Julie Grutzmacher, MSW, MPH, Director of National Collaboration and Engagement, Prevent Blindness	
Mary Elizabeth Hartnett, MD, Distinguished Professor in Ophthalmology and Visual Sciences, Moran Eye Center, University of Utah	
James F. Jorkasky, MBA, Executive Director, National Alliance for Eye and Vision Research	
Sarah Wells Kocsis, MBA, (Moderator), Vice President of Public Policy, Society for Women’s Health Research	
Laura M. Periman, MD, Founder and Director, Dry Eye Services and Clinical Research, Periman Eye Institute	
Erin M. Shriver, MD, FACS, Clinical Professor, Jim O’Brien Gross and Donnita Gross Chair of Ophthalmology, University of Iowa	
Nora M. Wong, MPH, Health Science Policy Analyst, National Eye Institute	

The SWHR Women’s Eye Health Working Group participated in literature reviews and discussions prior to the roundtable to identify priority topics in eye health research and care to address in the interdisciplinary setting. The roundtable consisted of a series of sessions that presented updates of research, clinical practice, and public health data concerning the various women’s eye conditions and diseases identified during the planning phase. An SWHR moderator used a discussion guide that was developed to facilitate an interactive dialogue that engaged participants in sharing quantitative and qualitative evidence to address the goals of the meeting. Based on the roundtable’s proceedings, the working group identified overarching themes concerning areas of need. The group then arrived at consensus agreement on the future directions to address emergent knowledge gaps in understanding the pathophysiology of eye diseases and barriers to clinical care that disproportionately affect women.

## Eye health disparities due to biological sex differences

### Age-related macular degeneration

Age-related macular degeneration (AMD) is the most common cause of irreversible vision loss among older adults (70 years and older), with an estimated 9 million Americans suffering with intermediate or advanced AMD [6]. Studies have reported conflicting results about prevalence in women and men [7]; however, older women are anticipated to bear a higher burden of disease due to longer life expectancy [8]. Age-adjusted studies have also found a higher burden of vision loss in women compared to men worldwide, suggesting that factors other than age are involved [9, 10]. Vision impairment caused by AMD results in increased functional disability, which can in turn increase risk for mental health problems such as clinical depression and anxiety, which are already more common in women [11].

Since the introduction of anti-vascular endothelial growth factor (anti-VEGF) therapy to treat AMD, vision loss can now be stabilized in over 90% of patients with neovascular AMD, which generally affects more women than men, regardless of race or ethnicity [7]. Anti-VEGF agents are used to inhibit the formation of new blood vessels behind the retina that are weak and ultimately hemorrhage materials that scar the retina and destroy macular cells. While current therapies can slow disease progression, there is still a need for curative or disease-reversal treatment options for AMD [12]. In addition, studies are needed to understand the causes of the burden of vision loss by gender, recognizing these may vary in regions throughout the world.

### Thyroid eye disease

According to the American Thyroid Association, 1 in 8 women will develop a thyroid disorder or disease, with women at increased risk after pregnancy and menopause. Graves’ ophthalmopathy, also known as thyroid eye disease (TED), is an autoimmune inflammatory condition of the eye and surrounding tissues. The higher prevalence in women is stark, with the disease affecting 16 women vs 3 men per 100,000 people annually [13]. The pathology of TED is complex; while it is often associated with the autoimmune thyroid disorder Graves’ disease, TED does not always coincide with thyroid activity or the treatment of underlying thyroid dysfunction. Clinicians have reported timing of initial symptoms to vary from up to 10 years before to 20 years after diagnosis of thyroid disease, [14, 15] making it challenging to diagnose TED independent of a pre-existing thyroid disorder.

Beyond thyroid dysfunction, the impacts of TED can be severe and disruptive to a patient’s quality of life [16]. Individuals can suffer from disfigurement, discomfort, visual impairment, and medication side effects—all of which are worsened by the long duration of treatment for this progressive disease. Due to differences in resources and availability of tools such as surgery, steroids, and symptom-managing medications, treatment approaches will vary depending on whether the provider is an endocrinologist, ophthalmologist, or other clinician. In 2020, the U.S. Food and Drug Administration (FDA) approved the first therapeutic for treatment of TED [17], which is administered intravenously. Patient responses to treatment can vary due to sex-based genetic and biochemical influences, as well as age, disease severity, and symptom severity. The advent of new therapies combined with earlier intervention and interdisciplinary clinical care could improve disease management and patient outcomes [18, 19].

### Dry eye disease

Dry eye disease (DED) is diagnosed in over 16 million individuals in the United States, with prevalence increasing with age, and an additional 2.5% of the population are estimated to experience symptoms without diagnosis [20]. The gender disparity in DED corresponds with age—for individuals over 50 years, women are twice as likely to have diagnosed DED compared to men. Women are often diagnosed at younger ages than men and experience more severe symptoms [21, 22]. Biological sex differences affect ocular structure, gene expression, and function that contribute to tear composition and output, as well as other characteristics that influence eye lubrication and health. While dry eye can occur in isolation, it is also associated with a host of comorbid conditions and autoimmune disorders, especially those that affect women, such as menopause, Graves’ disease, and chronic pain [22]. To improve patient quality of life, it is important that DED is identified early and treated by an appropriate eye care specialist. Educating providers and the public about this highly prevalent disease and its symptoms is essential to accomplishing this goal.

### Migraine

Migraine is a chronic neurological disease that affects more than 14% of the adult population worldwide [23]. In the United States, cumulative lifetime migraine incidence is 43% for women and only 18% for men [24]. Hormones and structural differences in the brain contribute to differing manifestations between the sexes. Women report longer migraine attacks, increased recurrence, greater disability, and longer recovery times [25]. Migraine is often associated with visual impairments including photophobia, visual aura, and transient vision loss. Visual quality of life has been found to be significantly reduced in patients with chronic and episodic migraine [26].

Studies have proposed correlations in the prevalence of migraine with DED. For example, one retrospective case–control study suggested that there is a 20% increased risk of DED diagnosis with an existing diagnosis of migraine [27]. For men, this association is more prevalent among those who are 55–64 years, whereas it is more pronounced in women of all ages. Dry eye also presents in migraine patients with greater presence of auras and longer disease and attack durations—symptoms which have been shown to disproportionately affect women [28]. Elucidating the causation and interplay of migraine and eye disease could provide better clinical strategies to treat comorbid conditions and improve visual quality of life for patients.

### Pregnancy and eye health

Pregnancy presents unique circumstances in a woman’s health journey, as the dramatic transformation the body undergoes often acts as a stress test for future health, including eye health [29]. During this time, women can develop eye disease that may or may not subside after pregnancy. For example, cellular immunity decreases during pregnancy, which may result in autoimmune diseases, such as TED, which can continue to worsen postpartum.

Another eye condition that can be induced or exacerbated by pregnancy is diabetic retinopathy (DR), a diabetes-related complication caused by damage to the blood vessels of the light-sensitive retinal tissue. If inadequately treated, DR can lead to blindness. Women with pre-existing diabetes mellitus can develop and are more likely to experience worsened DR during pregnancy [30]. In addition, a pregnant woman who has gestational diabetes is at risk for developing DR. The mechanism of DR progression in pregnancy is not fully understood, but studies have associated DR severity in early pregnancy with adverse health outcomes in the infant [31].

Fluid retention is a common occurrence during pregnancy, but it can also affect the eye’s corneal thickness and shape, resulting in visual disturbances. When presenting with high blood pressure and protein in the urine, these signs can sometimes indicate preeclampsia or eclampsia. (Pre)eclampsia-related retinal changes and dysfunction can lead to blurred or double vision, sudden transient vision loss, and flashing lights [32]. Visual symptoms occur in 25% of women with severe preeclampsia and 50% of patients with eclampsia [33]. Preeclampsia is more common in patients who have diabetes, which poses additional risk for comorbidity with DR [34]. The high blood pressure associated with preeclampsia can also contribute the retinal findings, such as vascular occlusions, serous retinal detachments, central serous retinopathy, and even cortical blindness. If women experience blurred or decreased vision, spots in vision, or color defects, they should be screened for preeclampsia. Efforts should be made to increase education and awareness of preeclampsia symptoms among both patients and providers to proactively prevent disease progression.

Opportunities exist within retinal imaging guidelines to increase screening for pregnant women, particularly for racial and ethnic minority patients who experience worse maternal health outcomes than their white counterparts [35]. Vision health should be considered for standard inclusion in pregnancy wellness visits. Furthermore, due to the multifaceted specialties of care needed to manage complex eye and other conditions during pregnancy, increased awareness and collaboration between ophthalmologists, optometrists, obstetricians, and internists could greatly enhance quality of care [36, 37].

## Gender inequities in eye health

Women experience societal pressures to keep up physical appearances, which often include the use of cosmetics and personal care products—an industry that is widely under-regulated and has had substantive unintended consequences on eye health, including contact dermatitis, bacterial infections, dry eye, and other serious conditions [38, 39]. For example, the use of contact lenses, especially cosmetic lenses, is higher in women [40], and is associated with higher rates of DED [41]. Retinoids in anti-aging creams applied around the eye have negative effects on the surrounding oil glands and can contribute to DED [42]. The application of eye makeup has been in practice for centuries and advancements in eyelash enhancing serums have gained popularity in recent years. The synthetic prostaglandins these products contain have also been linked to symptoms of dry eye and meibomian gland dysfunction [43]. Since many of these lash-lengthening products and dyes are sold over the counter, they are not subject to the same FDA regulatory and reporting standards required of medical compounds. Users, most often women, are at the mercy of potentially undisclosed ingredients, warnings, and/or side effects.

Society also imposes responsibilities and expectations on women to prioritize others’ well-being and health over their own. Women typically assume the role of family caregiver for children, partners, and aging parents [44]. This dynamic can leave women neglecting their own health needs and self-care. Health care burdens facing women are further exacerbated by disasters and health crises. The COVID-19 pandemic, for example, has skewed work-life balances and increased demands at home that have left women taking on additional tasks of caregiving, home-school teaching, career development, and entrepreneurship [45]. Unemployment during the pandemic has also disproportionately affected women—especially women of color—resulting in loss of employment-based health insurance and potentially longer term repercussions in gender inequality [46].

The Flatten Inaccessibility Survey provides insight into the disadvantages that individuals who are blind or have low vision faced early on the COVID-19 pandemic [47]. Although 30% of participants reported utilizing telehealth for doctor visits, 21% of participants indicated that the telehealth platform was not easily accessible. More female than male respondents expressed concerns about maintaining social distance in public due to their vision impairment (45% vs 18%, respectively), accessing a health care facility if they or a family member had severe symptoms (55% vs 25%), and experiencing inadequate caregiver assistance in a hospital setting (40% vs 20%). These valid concerns of women and individuals with eye health challenges warrant continued attention during and after the pandemic.

## Discussion

In 2016, the National Academies of Sciences, Engineering, and Medicine commissioned a report [48] that explored how population health approaches can provide frameworks and strategies to improve eye health, equity, and quality of life. The report’s recommendations include increasing public awareness, generating evidence to inform policy, promoting community action, and enhancing health capacity. Similarly, the World Health Organization released a *World report on vision* in 2019, proposing an integrated people-centered eye care approach to address persistent challenges and inequalities in health care delivery coordinated across different levels and throughout the life-course [49]. To advance the field and improve the understanding and treatment of eye health conditions across sex and gender, an integrative and systems-level perspective must be incorporated to implement changes in research approaches, care access and delivery, education, and partnerships (Table 2). Furthermore, the National Institute on Minority Health and Health Disparities (NIMHD) Research Framework serves as a guide that considers various domains of influence on a patient’s health journey throughout their life-course: biological, behavioral, physical/built environment, sociocultural environment, and health care system [50].

**Table 2:** Priority areas for women’s eye health research, care, and education

1.Adopting a research framework to guide future work that incorporates sex and gender lenses to investigate health disparities and inequities in women’s eye health care and disease
2.Using population health approaches to develop integrative systems to reduce disparities and improve overall eye health care for women at the individual, community, and systems levels
3.Increasing patient and provider education and awareness by standardizing medical curricula and expanding partnerships between eye care providers (e.g., ophthalmologists, optometrists), researchers, and non-vision providers to incorporate women’s eye health into mainstream health

### Expanding the research framework

Expanding the current research framework is essential to guide system innovations toward a population health approach to addressing women’s eye health vulnerability and disparities. Current eye health research is largely concentrated in clinical research, but evidence-based population health methods are needed to promote equitable awareness and treatments. Using the NIMHD research framework, sex and gender should also be prioritized for guidance on research questions and outcomes data analyses. The role of sex as a biological variable (SABV) in genetics, hormones, environmental exposures, and comorbidities should be investigated and data should be disaggregated by sex. Gender-based research should focus on the roles of behavior, differential access to care, cultural norms, and environmental influences on eye health disparities and inequities. This requires incorporating clearly defined SABV and gender standards and targets in research proposals and communications, which is now required by the National Institutes of Health, but is not currently a regular mandate by many funding organizations [51]. In 2020, SWHR released a position statement on the inclusion of SABV in research [52] recommending that researchers not only include both sexes within studies, but to also ensure that they have sufficient analytical power to return statistically significant outcomes when conducting sex-disaggregated data analyses. Furthermore, the importance of sex and gender inclusion in research and clinical studies, as well as appropriate training to do so, should be emphasized for all health professionals, not just researchers.

### Improving access through telehealth

Advances in health technology have created opportunities to increase access, diagnosis, and quality of vision care at more affordable costs for individuals across socioeconomic groups [53]. Before the COVID-19 pandemic, women were more likely to choose telemedicine compared to men, and some studies have further indicated that more women have presented as new patients during the health crisis, suggesting that maintaining expanded access to telehealth services post-pandemic would substantially benefit women [54]. The U.S. Department of Health and Human Services has relaxed its policies to enable increased access to telemedicine during the COVID-19 pandemic, and many private insurance providers followed suit. Thus, telehealth has made eye health appointments more convenient for many, especially women who may not have time to visit the doctor due to factors such as household responsibilities and transportation barriers. Comprehensive, community-based screening programs that combine telehealth services with ocular imaging tools and are stationed in centrally accessible locations for low-income or vulnerable populations (e.g., community centers, schools, or public housing communities) increase affordability and continuity of access to much-needed basic vision screening and care [55, 56]. It is imperative, however, that telehealth technology adheres to accepted standards, including web content accessibility guidelines recommended by international consortia such as W3C, to ensure that women and individuals of all visual abilities can utilize them effectively. In creating a sustainable telemedicine system for eye health, patient advocates should be consulted to provide critical guidance toward addressing design and implementation barriers to care, particularly experienced by female eye health patient populations.

### Provider and patient education

Improving education efforts at the patient and provider levels is essential to progressing the field of women’s eye health. A survey of 135 member institutions of the Association of University Professors of Ophthalmology and medical schools found that the majority of their respective programs provided preclinical exposure to ophthalmology, while clinical exposure varied widely—from interest groups to required clinical rotations [57]. Because of the interconnectedness of eye health to various other health conditions, ophthalmic training should be more standardized in medical school curricula. Non-eye health specialists—particularly primary care providers, pediatricians, and women’s health providers—should also be educated about risk factors and early indicators of eye health issues, eye screening recommendations, and pregnancy-related eye conditions to improve care and reduce inequities for women. In addition, information on SABV and gender differences and their broader implications in eye health should be incorporated during rotations and specialty training. Expanded awareness of how sex and gender influence eye health and care will better equip providers to assist patients in holistically understanding their eye health.

Building patient awareness and education about eye health aids in behavioral adjustments that could positively impact women’s eye health. Women should be informed of the potential eye health risks throughout the life-course, including issues associated with pregnancy (if part of their life plan) and menopause. Social media and mobile health apps provide accessible avenues to provide educational resources to patients and providers. These platforms also offer opportunities to connect with communities of support along the patient journey. When education efforts are implemented across the patient and provider spectrum, there is optimal opportunity for collaborative goal-setting and improved quality of care for all women.

### Interdisciplinary collaborations

The multi-level population health approach to improving women’s eye health leverages opportunities for partnerships and collaboration amongst eye health providers, non-ophthalmic providers, population health experts, and community stakeholders to create more holistic and comprehensive solutions to barriers to adequate research funding, access to quality care, and patient and provider education. By engaging stakeholders who are specialized for different populations and sectors of interest, efforts can build upon existing relationships and structures within communities. For example, if early childhood educators are aware of vision problems and how this can hinder development and behavior, attention can be given to children with vision issues to prevent them from falling behind academically. Conversely, older women should also be educated on eye health needs, as their risk increases for age-related eye diseases. Public health professionals, senior care specialists, and minority health specialists have the opportunity to integrate eye health education and early detection strategies into established programs that can target diverse populations. Many national nonprofit organizations and professional societies have established networks that can assist with promoting education among health care providers, advancements in research, building public awareness, and advocacy with policymakers. Fostering new cross-sector partnerships and enhancing existing ones between state and federal agencies, non-vision researchers (e.g., economists, gerontologists), and public and private organizations would also facilitate system-wide progress.

## Perspectives and significance

Various biological and social determinants of health contribute to eye health disparities and inequities for women. The solution is not necessarily to create a new system, but to adapt current systems to better address eye disease burden in women. Issues must be targeted through a multifaceted, systems-level approach that integrates women’s eye health as a priority in research, education, and collaboration across individual, clinical, and community-based settings. Finally, elevating the sex and gender lens in eye health research and care is key to advancing our understanding of eye health disparities among women and how these inequities can be rectified.

## Data Availability

Not applicable.

## Abbreviations

AMD: Age-related macular degeneration
DED: Dry eye disease
DR: Diabetic retinopathy
FDA: United States Food and Drug Administration
NIMHD: National Institute on Minority Health and Health Disparities
SABV: Sex as a biological variable
SWHR: Society for Women’s Health Research
TED: Thyroid eye disease

## References

[1] Fast Facts of Common Eye Disorders | CDC. 2020 [cited 2021 Feb 25]. https://www.cdc.gov/visionhealth/basics/ced/fastfacts.htm. Accessed 25 Feb 2021.

[2] Varma R, Vajaranant TS, Burkemper B, Wu S, Torres M, Hsu C (2016). Visual impairment and blindness in adults in the United States. JAMA Ophthalmol.

[3] Scott AW, Bressler NM, Ffolkes S, Ba M, Wittenborn JS, Jorkasky J (2016). Public attitudes about eye and vision health. JAMA Ophthalmol.

[4] Wizemann TM, Pardue M-L, Institute of Medicine. Exploring the Biological Contributions to Human Health: Does Sex Matter?. In: Wizemann TM, Pardue M-L, eds. Washington, DC: National Academies Press; 2001. pp 288. 25057540

[5] Clayton JA, Davis AF. Sex/gender disparities and women’s eye health. Vol. 40, Current Eye Research. Informa Healthcare; 2015. p. 102–9. 10.3109/02713683.2014.986333 25548854

[6] Chen Y, Bedell M, Zhang K (2010). Age-related macular degeneration: Genetic and environmental factors of disease. Mol Interventions.

[7] Yonekawa Y, Miller J, Kim I (2015). Age-related macular degeneration: advances in management and diagnosis. J Clin Med.

[8] Cimarolli VR, Casten RJ, Rovner BW, Heyl V, Sörensen S, Horowitz A. Anxiety and depression in patients with advanced macular degeneration: Current perspectives. Vol. 10, Clinical Ophthalmology. Dove Medical Press Ltd; 2016. p. 55–63. 10.2147/OPTH.S80489 PMC4699633 26766899

[9] Adelson JD, Bourne RRA, Briant PS, Flaxman SR, Taylor HRB, Jonas JB (2021). Causes of blindness and vision impairment in 2020 and trends over 30 years, and prevalence of avoidable blindness in relation to VISION 2020: the Right to Sight: an analysis for the Global Burden of Disease Study. Lancet Glob Health.

[10] Bourne R, Steinmetz JD, Flaxman S, Briant PS, Taylor HR, Resnikoff S (2021). Trends in prevalence of blindness and distance and near vision impairment over 30 years: an analysis for the Global Burden of Disease Study. Lancet Glob Health.

[11] National Institute of Mental Health. NIMH » Women and Mental Health. https://www.nimh.nih.gov/health/topics/women-and-mental-health/index.shtml. Accessed 15 Mar 2021.

[12] Pugazhendhi A, Hubbell M, Jairam P, Ambati B. Neovascular macular degeneration: A review of etiology, risk factors, and recent advances in research and therapy. Vol. 22, International Journal of Molecular Sciences. MDPI AG; 2021. p. 1–25. 10.3390/ijms22031170 PMC7866170 33504013

[13] Weiler DL (2017). Thyroid eye disease: a review. Clin Exp Optomet.

[14] Douglas RS, Gupta S. The pathophysiology of thyroid eye disease: Implications for immunotherapy. Vol. 22, Current Opinion in Ophthalmology. NIH Public Access; 2011. p. 385–90. 10.1097/ICU.0b013e3283499446 PMC3512192 21730841

[15] Khoo DH, Eng PH, Ho SC, Tai ES, Morgenthaler NG, Seah LL (2000). Graves’ ophthalmopathy in the absence of elevated free thyroxine and triiodothyronine levels: prevalence, natural history, and thyrotropin receptor antibody levels. Thyroid.

[16] Kahaly GJ, Petrak F, Hardt J, Pitz S, Egle UT (2005). Psychosocial morbidity of Graves’ orbitopathy. Clin Endocrinol.

[17] Douglas RS, Kahaly GJ, Patel A, Sile S, Thompson EHZ, Perdok R (2020). Teprotumumab for the treatment of active thyroid eye disease. N Engl J Med.

[18] Dolman PJ (2018). Grading severity and activity in thyroid eye disease. Ophthalmic Plast Reconstr Surg.

[19] Wang Y, Patel A, Douglas RS. Thyroid eye disease: How a novel therapy may change the treatment paradigm. Vol. 15, Therapeutics and Clinical Risk Management. Dove Medical Press Ltd.; 2019. p. 1305–18. 10.2147/TCRM.S193018 PMC6858302 31814726

[20] Farrand KF, Fridman M, Stillman IÖ, Schaumberg DA (2017). Prevalence of diagnosed dry eye disease in the united states among adults aged 18 years and older. Am J Ophthalmol.

[21] Vehof J, Sillevis Smitt-Kamminga N, Nibourg SA, Hammond CJ (2018). Sex differences in clinical characteristics of dry eye disease. Ocular Surface.

[22] Matossian C, McDonald M, Donaldson KE, Nichols KK, Maciver S, Gupta PK. Dry eye disease: Consideration for women’s health [Internet]. Vol. 28, Journal of Women’s Health. Mary Ann Liebert Inc.; 2019 [cited 2021 Feb 25]. p. 502–14. 10.1089/jwh.2018.7041 PMC6482917 30694724

[23] Schroeder RA, Brandes J, Buse DC, Calhoun A, Eikermann-Haerter K, Golden K (2018). Sex and gender differences in migraine - evaluating knowledge gaps. J Women’s Health.

[24] Stewart WF, Wood C, Reed ML, Roy J, Lipton RB (2008). Cumulative lifetime migraine incidence in women and men. Cephalalgia.

[25] Vetvik KG, MacGregor EA (2017). Sex differences in the epidemiology, clinical features, and pathophysiology of migraine. Lancet Neurol.

[26] Hanson LL, Ahmed Z, Katz BJ, Warner JEA, Crum AV, Zhang Y (2018). Patients with migraine have substantial reductions in measures of visual quality of life. Headache.

[27] Ismail OM, Poole ZB, Bierly SL, Van Buren ED, Lin FC, Meyer JJ (2019). Association between dry eye disease and migraine headaches in a large population-based study. JAMA Ophthalmol.

[28] Celikbilek A, Adam M (2015). The relationship between dry eye and migraine. Acta Neurol Belg.

[29] Kalogeropoulos D, Sung VCT, Paschopoulos M, Moschos MM, Panidis P, Kalogeropoulos C (2019). The physiologic and pathologic effects of pregnancy on the human visual system. J Obstetr Gynaecol..

[30] Kaaja R, Loukovaara S (2007). Progression of retinopathy in type 1 diabetic women during pregnancy. Curr Diabetes Rev.

[31] Klein BEK, Horak KML, Meuer SM, Mosher AE, Ewen AF, Danforth LG (2019). Retinal vessel diameters confound the relationship of pregnancy to retinopathy and infant outcomes in T1D. J Diabetes Compl.

[32] Roos NM, Wiegman MJ, Jansonius NM, Zeeman GG (2012). Visual disturbances in (pre)eclampsia. Obstetr Gynecolo Surv.

[33] Alizadeh Ghavidel L, Mousavi F, Bagheri M, Asghari S (2018). Preeclampsia induced ocular change. Int J Women’s Health Reprod Sci.

[34] Weissgerber TL, Mudd LM (2015). Preeclampsia and diabetes. Curr DiabRep.

[35] Nsiah-Kumi P, Ortmeier SR, Brown AE (2009). Disparities in diabetic retinopathy screening and disease for racial and ethnic minority populations—a literature review. J Natl Med Assoc.

[36] Mohammadi SF, Letafat-Nejad M, Ashrafi E, Delshad-Aghdam H (2017). A survey of ophthalmologists and gynecologists regarding termination of pregnancy and choice of delivery mode in the presence of eye diseases. J Curr Ophthalmol.

[37] Bohrer J, Ehrenthal DB (2015). Other adverse pregnancy outcomes and future chronic disease. Semin Perinatol.

[38] Califf RM, McCall J, Mark DB (2017). Cosmetics, regulations, and the public health understanding the safety of medical and other products. JAMA Internal Med.

[39] Cornell EM, Janetos TM, Xu S (2019). Time for a makeover-cosmetics regulation in the United States. J Cosmet Dermatol.

[40] Berenson AB, Hirth JM, Chang M, Merkley KH (2019). Knowledge and use of cosmetic contact lenses among reproductive-age women. J Women’s Health.

[41] Nichols JJ, Sinnott LT (2006). Tear film, contact lens, and patient-related factors associated with contact lens-related dry eye. Invest Ophthalmol Vis Sci.

[42] Lee BS, Kabat AG, Bacharach J, Karpecki P, Luchs J (2020). Managing dry eye disease and facilitating realistic patient expectations: A review and appraisal of current therapies. Clin Ophthalmol.

[43] Wang MTM, Craig JP (2018). Investigating the effect of eye cosmetics on the tear film: current insights. Clin Optomet..

[44] Chappell NL, Dujela C, Smith A (2015). Caregiver well-being: intersections of relationship and gender. Res Aging.

[45] Connor J, Madhavan S, Mokashi M, Amanuel H, Johnson NR, Pace LE (2020). Health risks and outcomes that disproportionately affect women during the Covid-19 pandemic: a review. Soc Sci Med.

[46] Alon T, Doepke M, Olmstead-Rumsey J, Tertilt M, Thank W, Bardoczy B, et al. This Time It’s Different: The Role of Women’s Employment in a Pandemic Recession. 2020 Aug. Report No.: 27660.

[47] Rosenblum PL, Chanes-Mora P, McBride CR, Flewellen J, Nagarajan N, Nave Stawasz R, et al. Flatten Inaccessibility: Impact of COVID-19 on Adults Who Are Blind or Have Low Vision in the United States. 2020.

[48] National Academies of Sciences E and M. Making Eye Health a Population Health Imperative: Vision for Tomorrow [Internet]. Teutsch SM, McCoy MA, Woodbury RB, Welp A, editors. Making Eye Health a Population Health Imperative. Washington, DC: National Academies Press; 2016. pp 586. 27656731

[49] World Health Organization. World report on vision. Vol. 214, World health Organization. Geneva; 2019.

[50] Alvidrez J, Castille D, Laude-Sharp M, Rosario A, Tabor D (2019). The National Institute on Minority Health and Health Disparities Research Framework. Am J Public Health.

[51] National Institutes of Health. NIH Policy and Guidelines on The Inclusion of Women and Minorities as Subjects in Clinical Research | grants.nih.gov. 2017.

[52] Society for Women’s Health Research. Inclusion of Sex as a Biological Variable in Research. 2020.

[53] Taylor CR, Merin LM, Salunga AM, Hepworth JT, Crutcher TD, O’Day DM (2007). Improving diabetic retinopathy screening ratios using telemedicine-based digital retinal imaging technology: the vine hill study. Diabetes Care.

[54] Reed ME, Huang J, Graetz I, Lee C, Muelly E, Kennedy C (2020). Patient characteristics associated with choosing a telemedicine visit vs office visit with the same primary care clinicians. JAMA Netw Open.

[55] Baker RS, Bazargan M, Bazargan-Hejazi S, Calderón JL (2005). Access to vision care in an urban low-income multiethnic population. Ophthal Epidemiol.

[56] Shahid K, Kolomeyer AM, Nayak NV, Salameh N, Pelaez G, Khouri AS (2012). Ocular telehealth screenings in an urban community. Telemed e-Health.

[57] Shah M, Knoch D, Waxman E (2014). The state of ophthalmology medical student education in the United States and Canada, 2012 through 2013. Ophthalmology.

